# Bayesian Averaging Evaluation Method of Accelerated Degradation Testing Considering Model Uncertainty Based on Relative Entropy

**DOI:** 10.3390/s24051426

**Published:** 2024-02-22

**Authors:** Tianji Zou, Wenbo Wu, Kai Liu, Ke Wang, Congmin Lv

**Affiliations:** 1 University of Chinese Academy of Sciences, Chinese Academy of Sciences, Beijing 100049, China; wuwenbo@csu.ac.cn (W.W.); lk@csu.ac.cn (K.L.); wangke@csu.ac.cn (K.W.); lvcongmin@csu.ac.cn (C.L.); 2 Technology and Engineering Center for Space Utilization, Chinese Academy of Sciences, Beijing 100094, China

**Keywords:** accelerated degradation testing, Bayesian evaluation, model uncertainty, model averaging, relative entropy

## Abstract

To evaluate the lifetime and reliability of long-life, high-reliability products under limited resources, accelerated degradation testing (ADT) technology has been widely applied. Furthermore, the Bayesian evaluation method for ADT can comprehensively utilize historical information and overcome the limitations caused by small sample sizes, garnering significant attention from scholars. However, the traditional ADT Bayesian evaluation method has inherent shortcomings and limitations. Due to the constraints of small samples and an incomplete understanding of degradation mechanisms or accelerated mechanisms, the selected evaluation model may be inaccurate, leading to potentially inaccurate evaluation results. Therefore, describing and quantifying the impact of model uncertainty on evaluation results is a challenging issue that urgently needs resolution in the theoretical research of ADT Bayesian methods. This article addresses the issue of model uncertainty in the ADT Bayesian evaluation process. It analyzes the modeling process of ADT Bayesian and proposes a new model averaging evaluation method for ADT Bayesian based on relative entropy, which, to a certain extent, can resolve the issue of evaluation inaccuracy caused by model selection uncertainty. This study holds certain theoretical and engineering application value for conducting ADT Bayesian evaluation under model uncertainty.

## 1. Introduction

Modern industries have a continually growing demand for long-life, high-reliability products. Accelerated testing technology subjects products to harsher conditions than their normal usage environment, thus accelerating the product’s failure or degradation process. This allows for the collection of lifespan or degradation data in a shorter period for reliability or lifespan evaluation [1]. Traditional ADT analysis methods are based on probability theory. Nelson, W.B. [2] studied lifetime distribution based on performance degradation, providing a comprehensive description of ADT, including its applied scope, test plans, statistical models, and data analysis methods. Meeker, W.Q. [3] provided a detailed exposition of degradation models, accelerated models, and failure-related models and analysis in ADT technology. However, with insufficient data from small sample sizes, these probabilistic-based ADT evaluation methods expose some flaws.

The Bayesian method can integrate ADT data from multiple sources, overcoming the limitations caused by small sample sizes [4]. Therefore, it has been greatly emphasized in recent years. Prakash, G. [5] presented two Bayesian hierarchical models: one utilizing the lifetime data and the other using structural health monitoring data for degradation modeling and reliability assessment of rolling element bearings. Pang, Z. [6] proposed a Bayesian model that differs from existing models by adopting non-conjugate prior distributions for random-effect parameters, which can obtain higher Remaining Useful Life estimation accuracy and less uncertainty. Fan, T.-H. [7] used Bayesian predictive analysis based on the inverse Gaussian process with conjugate priors to deduce the failure time inference, which is associated with the degradation model, and its goodness-of-fit test is suggested from a complete Bayesian perspective and can also be used for other degradation models with random effects. However, the ADT Bayesian evaluation method also has inherent flaws and shortcomings. When dealing with the data collected through ADT, one may face the issue of having multiple options for evaluation models. Insufficient small-sample data and an incomplete understanding of the product’s degradation mechanism can both lead to incorrect model selection [8], resulting in inaccurate product assessments. Hence, discussing the issue of model uncertainty is of paramount importance for the ADT Bayesian method.

In the modeling area of ADT, there has been substantial research in recent years. In the field of degradation modeling, Ye, Z.S. [9] provided a detailed review of commonly used stochastic processes and their applications in degradation modeling, design of experiments, and aging stage assessment. Chhikara, R.S. [10] and Whitmore, G.A. [11] conducted in-depth research on degradation models based on Brownian motion. Bagdonavicius, V. [12] proposed a performance degradation model based on the gamma process. Lawless, J. [13] used a gamma process with covariates and random effects to describe the performance degradation process during the implementation of ADT. Zhang, Y. [14] established a reliability evaluation model based on the Normal-Poisson process on the condition of small sampling testing, combining the characteristics of aerospace product performance degradation mechanisms. Tsai, C.-C. [15] analyzed the impact on the product’s mean time to failure values after incorrectly defining gamma and Wiener processes as a degradation model. In the field of accelerated modeling, the earliest research was on the single-stress accelerated model [16]. Currently, widely recognized and accepted single-stress accelerated models include the Arrhenius model, which describes temperature-induced performance degradation [17]; the Eyring model, used to describe chemical aging [18]; and the power law [19] and exponential law [20] models for describing the relationship between electrical stress and product life, etc. Pan, Z. [21] considered the issue of model uncertainty in the step-stress ADT, compared the differences between the Arrhenius model and the Power model, and applied the Bayesian Markov chain Monte Carlo (MCMC) method to obtain the maximum likelihood estimation for key parameters.

Currently, researchers have proposed various solutions to address the issue of model uncertainty, with model averaging being one commonly used approach among them. Model averaging involves researching how to combine estimates or predictions from different models using certain weights [22]. In Bayesian statistical theory, relative entropy measures the distance between the prior distributions and the posterior distributions, representing the information obtained from sample data and the model, often referred to as information gain (IG) [23]. Based on Lindley’s proposal in reference [24], it is a viable option to choose a model analysis method that considers using IG as a criterion to judge the quality of models. Busetto, A.G. [25] defined the expected IG obtained from the sample data as the choice of the Bayesian model distribution. This implies that a model offering greater information gain under the same sample data also suggests a superior model-fitting effect. Hence, it can be considered to use relative entropy values as a reference for model weights and establish a new model averaging theory. This can serve as a foundation for analyzing model uncertainty in ADT Bayesian evaluation.

In summary, to address the issue of model uncertainty in ADT Bayesian evaluation, it is essential to quantitatively assess the contribution of different models to the evaluation process using the same sample data. By following the guiding principle of model averaging, it is possible to use the values of the IG as the weights for individual models participating in model averaging. The structure of this paper is as follows: Section 2 introduces the ADT model, including the degradation model and the acceleration model, and establishes a unified ADT Bayesian evaluation model and evaluation framework. In Section 3, the average evaluation method based on relative entropy is introduced, which mainly includes how to obtain model weights through relative entropy and the process of evaluation. Section 4 introduces a simulation case: using different ADT Bayesian models to evaluate the same simulated sample data, and conducting ADT Bayesian average evaluation based on relative entropy as model weights. By comparing the evaluation results of the average evaluation method with those of traditional separate Bayesian models, as well as the true evaluation results represented by the simulation assumptions, the feasibility of the Bayesian averaging evaluation method based on relative entropy is demonstrated. Section 5 provides a conclusion and an outlook for future research.

## 2. Modeling of ADT Bayesian Evaluation

### 2.1. Bayesian Inference

In the traditional Bayesian framework, statistical inference typically involves three types of information: (1) Population information, which includes information about the overall distribution of the data. The population information is hidden in the models. (2) Sample information, which is obtained from drawing a sample. (3) Prior information, which refers to information obtained before sampling, is typically derived from historical data and empirical knowledge. Statistical inference based on these three types of information is known as Bayesian statistics. According to the Bayesian theorem, the Bayesian equation can be written as

(1) $$\pi \left(\theta |y\right)=\frac{f\left(y|\theta \right)\cdot \pi \left(\theta \right)}{m\left(y\right)},$$

where *π*(***θ***|*y*) is the posterior distribution of parameters ***θ*** given the observed data *y*. *f*(*y*|***θ***) is referred to as the likelihood function, representing the probability distribution of *y* given parameters ***θ***. *π*(***θ***) is the prior distribution of prior parameters ***θ***, describing ***θ*** in the absence of sample information. *m*(*y*) is the marginal density function of *y*, which contains no information about parameters ***θ***. The posterior distribution *π*(***θ***|*y*) is the result that encompasses all information about the parameter ***θ*** from the prior, population and sample information. The marginal density function *m*(*y*) can be represented by the following equation:

(2) $$m\left(y\right)=\int_{\Theta }f\left(y|\theta \right)\pi \left(\theta \right)d\theta ,$$

where Θ represents the space of values for the parameters ***θ***. To simplify the equation, we will omit Θ in the following text. Meanwhile, *m*(*y*) does not depend on ***θ*** and serves as an essential “normalization” constant. According to the reference [26], *f*(*y*|***θ***) can also be referred to as the likelihood function representing the probability distribution of ***θ*** given parameter *y*, denoted as *l*(***θ***|*y*), as follows:

(3) $$l\left(\theta |y\right)=f\left(y|\theta \right)=\prod_{i=1}^{n}f\left(y_{i}|\theta \right).$$

In the Bayesian equation, the likelihood function plays a crucial role. It revises the prior information about ***θ*** based on the observed data *y*. Thus, the posterior distribution can be considered as the result of adjusting the prior distribution with the combined population information and sample information.

### 2.2. Models of ADT

#### 2.2.1. Degradation Model

Stochastic processes have a time-dependent structure, making it possible to characterize the influence of random factors in the degradation process. Therefore, they are used to describe the degradation models of products over time [27]. Currently, there has been extensive research on these stochastic processes, and they have found wide applications in the modeling of various fields. This paper proposes a unified stochastic degradation model, which contains three types of stochastic processes, namely the Wiener process, gamma process, and lognormal process.

For a traditional constant-stress ADT case [28], *Y*(*t*) represents the degradation process of a product’s performance parameter. *Y*(*t_ijk_*) represents the degradation data value at detection time *t_ijk_*, where *i* = 1,2,…,*l* denotes the number of accelerated stress levels, *j* = 1,2,…,*m_i_* represents the number of samples tested at the *i*-th accelerated stress level, and *k* = 1,2,…,*n_ij_* denotes the number of detections for the *j*-th sample at the *i*-th stress level [29]. Δ*y*(*t_ijk_*) = *Y*(*t_ijk_*) − *Y*(*t_ij_*_(*k*−1)_) is defined as the degradation increment at time *t_ijk_* and Δ(*t_ijk_*) = ρ(*t_ijk_*) − ρ(*t_ij_*_(*k*−1)_) is defined as the time increment, where ρ(*t*) is a function of detection time, which is defined as ρ(*t*) = *t* in this paper, representing a linear function of time. In ADT analysis, *y*(*t*) = Δ*y*(*t_ijk_*) is defined as the new form of data. It is typically assumed that the degradation increment *y*(*t*) follows a unified stochastic process of independent increments, denoted as

(4) $$y\left(t\right)\sim f\left(y\left|\alpha \left(t\right),\beta \left(t\right)\right.\right),$$

which characterize the product’s performance degradation over time. Here, *α*(*t*) represents the location or scale parameter of the stochastic process, and *β*(*t*) represents the shape parameter of the stochastic process. The expected value and variance of *y*(*t*) are proportional to the time function Λ(*t*), which is given by

(5) $$E\left(y\left(t\right)\right)=u\cdot \Lambda \left(t\right),$$

(6) $$\mathrm{Var}\left(y\left(t\right)\right)=\sigma^{2}\cdot \Lambda \left(t\right).$$

where Λ(*t*) is the transformation model for the time increment Δ*t_ijk_*. The parameter *u* represents the degradation rate of the product’s degradation process, which is related to the product’s stress level *s*. *u* and *σ* can be expressed in terms of the parameters *α*(*t*), *β*(*t*), and vice versa; *α*(*t*) and *β*(*t*) can be expressed in terms of the parameters *u* and *σ*. Unified stochastic processes help with model uncertainty analysis and averaging evaluation.

The Wiener process is one of the most basic, simple, and important stochastic processes [30]. When the degradation process model of product performance follows the Wiener process, it means *y*(*t*) follows a normal distribution with location parameter *α*(*t*) = *u·*Λ(*t*) and shape parameter *β*(*t*) = *σ*^2^*·*Λ(*t*), i.e., *f*_N_(*y*|*α*(*t*),*β*(*t*))~*N*(*u·*Λ(*t*),*σ*^2^*·*Λ(*t*)) [31]. The probability density function (PDF) of *y*(*t*) is shown in Equation (7).

(7) $$y\left(t\right)\sim f_{N}\left(y|\alpha \left(t\right),\beta \left(t\right)\right)=\frac{1}{\sigma \sqrt{2\pi \cdot \Lambda \left(t\right)}}\exp\left\{-\frac{{\left[y-u\cdot \Lambda \left(t\right)\right]}^{2}}{2\sigma^{2}\cdot \Lambda \left(t\right)}\right\}.$$

Through the PDF, the likelihood function *l*(·) of the Wiener process in Equation (3) can be obtained. The logarithmic form of the likelihood function *L*_N_(·) is shown in Equation (8).

(8) $$L_{N}\left(u,\sigma |y\right)=\sum_{i=1}^{l}\sum_{j=1}^{m_{i}}\sum_{k=1}^{n_{ij}}\left\{-\ln\sigma -\frac{1}{2}\ln2\pi \cdot \Lambda \left(t_{ijk}\right)-\frac{{\left(y_{ijk}-u\cdot \Lambda \left(t_{ijk}\right)\right)}^{2}}{2\sigma^{2}\Lambda \left(t_{ijk}\right)}\right\}.$$

The gamma process is a type of stochastic process with non-negative increments, which is well suited for describing strictly monotonic degradation processes, such as wear and crack growth [29]. When the degradation model follows the gamma process, the degradation increment *y*(*t*) is subject to the gamma distribution with the scale parameter *α*(*t*) = *u*/*σ*^2^ and the shape parameter *β*(*t*) = *u*^2^*·*Λ(*t*)/*σ*^2^. The PDF of *y*(*t*) is seen in Equation (9).

(9) $$y\left(t\right)\sim f_{Ga}\left(y\left|\alpha \left(t\right),\beta \left(t\right)\right.\right)=\frac{{\left(u/\sigma^{2}\right)}^{u^{2}\cdot \Lambda \left(t\right)/\sigma^{2}}}{\Gamma \left(u^{2}\cdot \Lambda \left(t\right)/\sigma^{2}\right)}y^{\frac{u^{2}\cdot \Lambda \left(t\right)}{\sigma^{2}}-1}\exp\left(-\frac{u\cdot y}{\sigma^{2}}\right),y>0.$$

Γ(·) in Equation (9) is the gamma function. The logarithmic form of the likelihood function *L*_Ga_(·) for the gamma process is expressed in Equation (10).

(10) $$L_{Ga}\left(u,\sigma |y\right)=\sum_{i=1}^{l}\sum_{j=1}^{m_{i}}\sum_{k=1}^{n_{ij}}\left\{\begin{array}{l} \frac{u^{2}\cdot \Lambda (t_{ijk})}{\sigma^{2}}\ln\left(\frac{u}{\sigma^{2}}\right)-\\ \ln\Gamma \left(\frac{u^{2}\cdot \Lambda (t_{ijk})}{\sigma^{2}}\right)+\left(\frac{u^{2}\cdot \Lambda (t_{ijk})}{\sigma^{2}}-1\right)\ln y_{ijk}-\frac{u\cdot y_{ijk}}{\sigma^{2}} \end{array}\right\}.$$

Meanwhile, this paper proposes an ADT Bayesian model based on the lognormal process. The lognormal process also has the characteristics of non-negative increments and is suitable for describing strictly monotonic degradation processes. Compared to the normal distribution, the lognormal distribution has more values in the upper tail in the long term, making it become one of the most common distribution models in reliability testing [32]. When the degradation model follows a lognormal process, ln(*y*(*t*)) follows a normal distribution. The location parameter *α*(*t*) can be expressed as

(11) $$\alpha \left(t\right)=\ln\left(\left(u^{2}\cdot \Lambda {\left(t\right)}^{2}\right)/\sqrt{u^{2}\cdot \Lambda {\left(t\right)}^{2}+\sigma \cdot \Lambda \left(t\right)}\right).$$

The shape parameter *β*(*t*) can be expressed as

(12) $$\beta \left(t\right)=\ln\left(1+\sigma^{2}/\left(u^{2}\cdot \Lambda \left(t\right)\right)\right).$$

Therefore, the PDF of *y*(*t*) is shown in Equation (13).

(13) $$\begin{array}{ll} y\left(t\right)\sim f_{Ln}\left(y|\alpha \left(t\right),\beta \left(t\right)\right) & =\frac{1}{y\cdot \sqrt{\ln\left(1+\sigma^{2}/\left(u^{2}\cdot \Lambda \left(t\right)\right)\right)\cdot 2\pi }}\times \\ & \exp\left\{-\frac{{\left[\ln y-\ln\left(\left(u^{2}\cdot \Lambda {\left(t\right)}^{2}\right)/\sqrt{u^{2}\cdot \Lambda {\left(t\right)}^{2}+\sigma \cdot \Lambda \left(t\right)}\right)\right]}^{2}}{2\cdot \ln\left(1+\sigma^{2}/\left(u^{2}\cdot \Lambda \left(t\right)\right)\right)}\right\},y>0 \end{array}.$$

The logarithmic form of the likelihood function *L*_Ln_(·) for the lognormal process is shown in Equation (14).

(14) $$L_{Ln}\left(u,\sigma |y\right)=\sum_{i=1}^{l}\sum_{j=1}^{m_{i}}\sum_{k=1}^{n_{ij}}\left\{\begin{array}{l} -\ln y_{ijk}-\frac{1}{2}\ln\left(\ln\left(1+\frac{\sigma^{2}}{u^{2}\cdot \Lambda \left(t_{ijk}\right)}\right)\cdot 2\pi \right)-\\ \frac{{\left[\ln y_{ijk}-\ln\left(u^{2}\cdot \Lambda {\left(t_{ijk}\right)}^{2}/\sqrt{u^{2}\cdot \Lambda {\left(t_{ijk}\right)}^{2}+\sigma \cdot \Lambda \left(t_{ijk}\right)}\right)\right]}^{2}}{2\cdot \ln\left(1+\sigma^{2}/\left(u^{2}\cdot \Lambda \left(t_{ijk}\right)\right)\right)} \end{array}\right\}.$$

#### 2.2.2. Accelerated Model

The accelerated model is used to describe the relationship between the accelerated stress *s* and the degradation rate *u* [33]. This paper considers only a single-stress accelerated model. There are widely recognized and accepted single-stress accelerated models including the Arrhenius relation, power law relation, exponential relation, etc. [34]. Through logarithmic transformations, the above accelerated models can be expressed simply using a linear equation, seen as

(15) $$\varphi \left(\mu_{i}\right)=a+b\cdot \varphi \left(s_{i}\right).$$

In Equation (15), *a* and *b* are two unknown parameters, *φ*(*u_i_*) represents a function of the degradation rate *u*, and *φ*(*s_i_*) is a function of the accelerated stress level *s_i_*. Unifying the unknown parameters of *a* and *b* facilitates conducting uncertainty analysis of model.

### 2.3. Evaluation Framework of ADT Bayesian Model

According to Equation (3), the likelihood function of different ADT models under the Bayesian evaluation framework is shown in Equations (8), (10) and (14), which can be simplified to the following unified expression:

(16) $$\ln\left(f\left(y|\theta \right)\right)=\ln\left(l\left(\theta |y\right)\right)=\sum_{i=1}^{l}\sum_{j=1}^{m_{i}}\sum_{k=1}^{n_{ij}}\ln\left(f\left(a,b,\sigma |y_{ijk}\right)\right).$$

**Figure 1:**
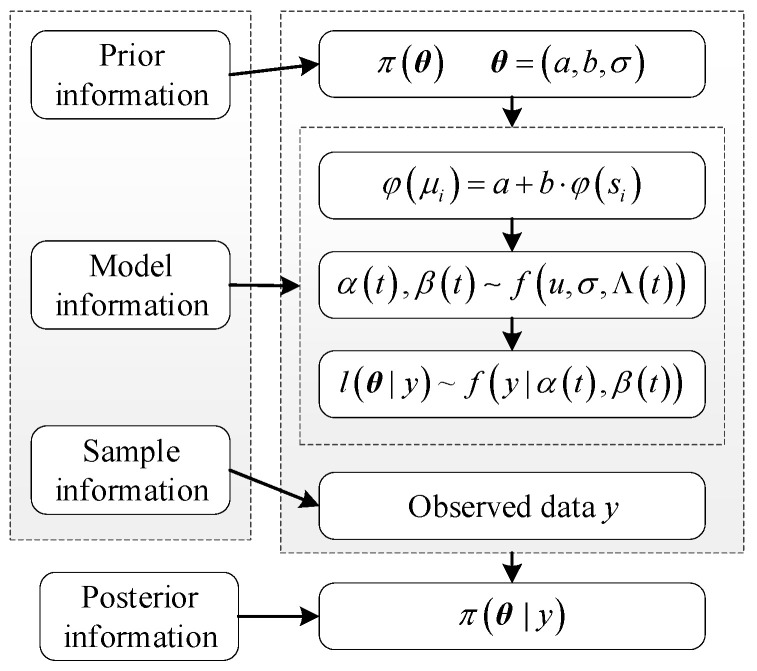
Framework of ADT Bayesian model.

The unknown parameters of the likelihood function, ***θ*** = (*a*,*b*,σ), represent the prior parameters. The framework of the ADT Bayesian model is illustrated in Figure 1.

One important thing about the evaluation method is obtaining the failure time (or first passage time) of a product. Assuming *d* is the failure threshold for product performance, it means that the product fails when *y*(*t*) − *d* < 0. In many cases, the models for failure can be approximated closely by accelerated test versions of Birnbaum–Saunders and inverse Gaussian distributions [35]. The framework of evaluation is quite general and allows for different degradation processes, including the Wiener process, gamma process, lognormal process, etc. The reliability function, which is the complementary cumulative distribution function (CDF) of the lifetime in general and also known as the survival function [36], of the first passage time is shown as

(17) $$R(t)=\Phi \left[\frac{d-u\cdot \Lambda (t)}{\sigma \sqrt{\Lambda (t)}}\right]-\exp\left(\frac{2u\cdot d}{\sigma^{2}}\right)\Phi \left[-\frac{d+u\cdot \Lambda (t)}{\sigma \sqrt{\Lambda (t)}}\right],$$

where Φ(·) is the standard normal CDF. When *u* is much larger than *σ*, the second part of Equation (17) can be neglected [37]. *y*(*t*) is approximately normally distributed with mean *u·*Λ(*t*) and variance *σ*^2^*·*Λ(*t*). The reliability function can be simplified as

(18) $$R\left(t\right)\approx \Phi \left[\frac{d-u\cdot \Lambda \left(t\right)}{\sigma \sqrt{\Lambda \left(t\right)}}\right]$$

According to [38], Λ(t) follows a two-parameter Birnbaum–Saunders distribution with respect to *τ* and *δ*; the reliability function expression can be converted to Equation (19), where *τ = σ*/$\sqrt{d\cdot \mu }$ and *δ* = *d*/*u.*

(19) $$R\left(t\right)≅\Phi \left[\frac{1}{\tau }\left({\left(\frac{\delta }{\Lambda \left(t\right)}\right)}^{1/2}-{\left(\frac{\Lambda \left(t\right)}{\delta }\right)}^{1/2}\right)\right].$$

In the framework of the ADT Bayesian method, *u·*Λ(*t*) = E(*Y*(*t*)|π(***θ***|*y*)) and *σ*^2^*·*Λ(*t*) *=* Var(*Y*(*t*)|π(***θ***|*y*)) represent the mean and variance of the product degradation under the posterior distribution. Equation (18) can be translated into the reliability function of the Bayesian assessment model, which can be expressed as

(20) $$R\left(t\right)\approx \Phi \left[\frac{d-E\left(Y\left(t\right)|\pi \left(\theta |y\right)\right)}{\sqrt{\mathrm{Var}\left(Y\left(t\right)|\pi \left(\theta |y\right)\right)}}\right].$$

Through reliability function of the unified ADT Bayesian model, the lifetime under normal stress conditions can be evaluated.

## 3. Averaging Method Based on Relative Entropy

### 3.1. Relative Entropy

**Figure 2:**
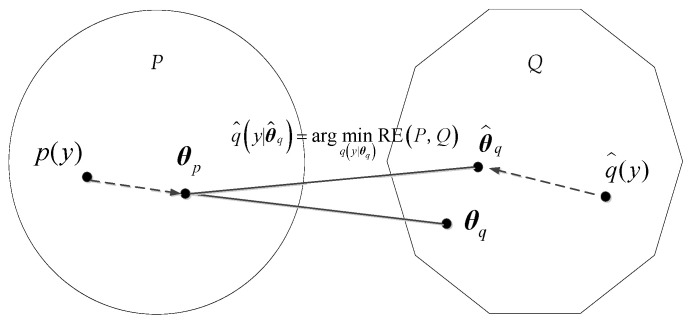
Relative entropy in model comparison.

Relative entropy is a measure of the asymmetry between two probability distributions *P* and *Q*. *P* represents the true distribution of data, while *Q* represents the theoretical or estimated model distribution of the data. Relative entropy can be used to measure the similarity between two probability distributions [39]. The relative entropy of probability distributions *P* and *Q* is defined as follows:

(21) $$\mathrm{RE}\left(P,Q\right)=\int_{-\infty }^{\infty }p\left(y\right)\cdot \log\frac{p\left(y\right)}{q\left(y\right)}dy,$$

where *p*(*y*) and *q*(*y*) represent the probability density functions of distributions *P* and *Q*, respectively, assuming that the model distributions *P* and *Q* have distribution parameters ***θ****_p_* and ***θ****_q_*, respectively. The optimal model *Q* is the one that minimizes the relative entropy. Figure 2 shows the diagram of relative entropy in model comparison.

In the Bayesian theory, relative entropy can be used as a measure of the IG in moving from a prior distribution to a posterior distribution [40]. In this paper, the information entropy contained in the prior distribution *π*(***θ***) under Bayesian model *M* is denoted as I_0_, shown as

(22) $$I_{0}\left(\theta \left|M\right.\right)=\int \pi \left(\theta \right)\log\pi \left(\theta \right)d\theta {=E}_{\theta }\left[\log\pi \left(\theta \right)\right],$$

where E***_θ_***(·) represents the expectation with respect to ***θ***, which is the prior parameter of *M*. The logarithms in these formulae are usually taken to base *e* for information to be measured. The information entropy contained in the posterior distribution *π*(***θ***|*y*) is denoted as I_1_, shown as

(23) $$I_{1}\left(\theta |y,M\right)=\int \pi \left(\theta \left|y\right.\right)\cdot \log\pi \left(\theta \left|y\right.\right)d\theta {=E}_{\theta }\left[\log\pi \left(\theta \left|y\right.\right)\right].$$

Therefore, the relative entropy between the prior distribution and the posterior distribution is defined as

(24) $$I\left(\theta |M\right)=I_{1}\left(\theta |y,M\right)-I_{0}\left(\theta |M\right)=E_{\theta }\left[\log\frac{\pi \left(\theta |y\right)}{\pi \left(\theta \right)}\right].$$

Based on Equation (1), the expression of relative entropy can be transformed into

(25) $$I\left(\theta |M\right)=E_{\theta }\left[\log\frac{f\left(y|\theta \right)}{m\left(y\right)}\right].$$

Since in most cases, obtaining the analytical expression of the posterior for direct computation is difficult, numerical simulation and calculation are commonly used methods for posterior computation. E***_θ_***[log *f*(*y*|***θ***)] can be calculated in both parameter space and sample space using Monte Carlo simulation methods through the likelihood function *f*(*y*|***θ***). The computation of the marginal likelihood function *m*(*y*) is an important research area in the Bayesian and Monte Carlo simulation fields. Laplace–Metropolis estimation can be employed for the asymptotic estimation of the marginal likelihood [41]. The calculation formula of *m*(*y*) is shown as follows:

(26) $$m(y)\approx {\left(2\pi \right)}^{D/2}\cdot {\left|\sum_{\theta }\right|}^{1/2}\cdot f(y|\bar{\theta })\cdot \pi (\bar{\theta }),$$

where $\overset{-}{\theta }$ and Σ***_θ_*** are the posterior mean and the variance–covariance matrix of the parameters, respectively. *D* is the dimension of the model parameter vector. The calculation formula can be expressed as

(27) $$\bar{\theta }=\frac{1}{R_{ML}}\cdot \sum_{g=1}^{R_{ML}}\theta_{g},$$

(28) $$\sum_{\theta }=\frac{1}{R_{ML}-1}\cdot \sum_{g=1}^{R_{ML}}\left(\theta_{g}-\bar{\theta }\right)\cdot {\left(\theta_{g}-\bar{\theta }\right)}^{T},$$

where *R_ML_* represents the number of parameter samples {***θ***_1_,…,$\theta_{R_{\mathrm{ML}}}$} sampled from the posterior distribution. Using the WinBUGS v.1.4.3 software and employing the MCMC sampling method can effectively solve this computational solution problem.

### 3.2. Weight of Model

The posterior distribution obtained by using Bayesian theory has acquired a new information entropy compared to the prior distribution through model information and sample information [42]. Therefore, the relative entropy values of the same sample data under different models are different. From the perspective of information entropy, relative entropy can quantify the IG of the model. Therefore, the relative entropy can be used as weights for the models participating in averaging evaluation. At the same time, relative entropy is not only related to models but also to prior distributions. Therefore, it is advisable to use non-informative prior distributions for relative entropy calculations to reduce the influence of prior distributions on weight calculations. By allocating weights to different models, an averaging evaluation method is established that considers model uncertainty.

Let ***M*** = (*M*_1_, *M*_2_,…, *M_v_*,...) (1 ≤ *v* ≤ r) represent the Bayesian evaluation model space composed of all possible individual models. *M_v_* represents the *v*-th model, and *ω_v_* is the weight assigned to *M_v_* based on relative entropy. For different models that depend on the same sample data *y*, their weights in the model averaging method are as follows:

(29) $$\omega_{v}=\frac{I_{v}\left(y,M_{v}\right)}{\sum_{v=1}^{r}I_{v}\left(y,M_{v}\right)}.$$

Due to the difficulty in obtaining analytical solutions for the integrated form of the likelihood function *f*(*y*|***θ***) after weight allocation, the MCMC method is provided to conduct evaluation based on relative entropy. This method involves extracting samples from the posterior distribution *π*(***θ_v_****|y*,*M_v_*) through WinBUGS software [41]. Then, assessment is conducted by using samples from the posterior parameters. Under this approach, the weights derived from relative entropy can be used as the weights of posterior parameters participating in the evaluation, which represent the contribution of different models to the model averaging method. Ultimately, the weight values affect the lifetime assessment results of the product under normal stress conditions. Based on the convolution formula of Gaussian distribution under the sample data *y*, the reliability function of the first-passage time based on relative entropy weights is

(30) $$\bar{R}\left(t|y\right)\sim N\left(\bar{u},{\bar{\sigma }}^{2}\right),$$

where $\overset{-}{u}$ and ${\bar{\sigma }}^{2}$ represent the mean and variance of degradation rate, respectively, after model weight allocation, expressed as

(31) $$\bar{u}=\sum_{v=1}^{r}\omega_{v}\cdot u_{v},$$

(32) $${\bar{\sigma }}^{2}=\sum_{v=1}^{r}\omega_{v}^{2}\cdot \sigma_{v}^{2}\;.$$

By using the WinBUGS software, *u_v_* and *σ_v_* can be obtained from the posterior distributions through MCMC sampling statistical methods. Using the model averaging evaluation method based on relative entropy can help reduce the impact of model uncertainty on evaluation results to some extent.

### 3.3. Averaging Evaluation Process

In this section, the process of conducting the Bayesian averaging evaluation method of ADT considering model uncertainty based on relative entropy has been reorganized.

(1) Constructing the set {*M_v_*} of the ADT models

To carry out the uncertainty analysis of the ADT Bayesian model, the first step is to select different degradation models of stochastic processes and accelerated models for cross-combination in order to build an evaluation model set ***M*** = {*M_v_*}.

(2) Bayesian modeling for each individual evaluation model

For each *M_v_* in the set of models, Bayesian modeling is implemented and the likelihood function *l*(***θ_v_****|y*,*M_v_*)) is established independently. The Doodle model can be built through WinBUGS software, which involves inputting the sample data, compiling the model, setting initial values, etc. [41].

(3) Setting of prior distribution *π*(***θ_v_****|M_v_*) for each Bayesian models

It is recommended to use uninformative prior distributions in the Bayesian calculation, such as uniform distribution within a certain range, which ensure the consistency of the prior entropy of different Bayesian evaluation models as much as possible.

(4) Inference of posterior distribution *π*(***θ_v_****|y*,*M_v_*)

Combining the sample data *y*, the samples of the posterior parameters are obtained after assessing the convergence of the posterior parameter through WinBUGS software. Then, a certain number of samples are discarded from the posterior parameter vector as the aging phase and a fixed number of samples are sampled for subsequent calculations.

(5) Calculation of relative entropy I(***θ***| *M_v_*) and model weights *ω_v_*

By adopting the Monte Carlo simulation method within the posterior parameter sample space, relative entropy is calculated. Based on the values of relative entropy, weights are assigned to each individual model, and an averaged model that considers model uncertainty is formed.

(6) Analysis of outcome

By sampling from different model parameter posterior distributions according to weight proportions, the integrated posterior distribution $\overset{-}{\pi }(\theta |y,M)$ is obtained. Then, this distribution is utilized for ADT evaluation and the results are analyzed.

## 4. An Illustrative Simulation Case

### 4.1. Simulation Data Declaration

To illustrate the effectiveness of the method proposed in this paper, this section employs simulated data as sample data to validate the theory of ADT Bayesian averaging evaluation considering model uncertainty based on relative entropy. The simulated data are generated by a specified model, which facilitates the analysis and comparison of results. The case study focuses on an SSADT, with the simulation model comprising a lognormal process for degradation modeling and an Arrhenius relation for accelerated modeling. The basic information settings for the illustrative simulation case are presented in Table 1.

**Figure 3:**
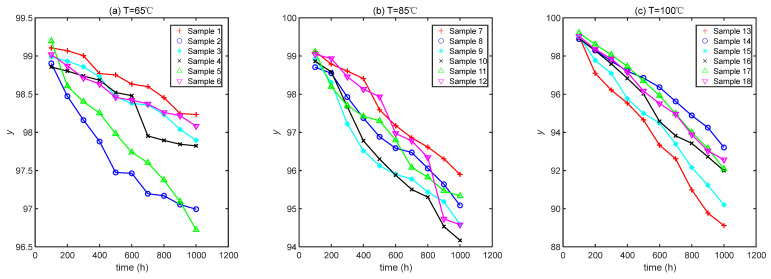
Degradation curves of simulation case.

Figure 3 shows the degenerate path of simulated data. The horizontal axis represents the detection time *t*, and the vertical axis represents the product performance *Y*(*t*). When the performance degradation exceeds 30, which means the failure threshold *d* = 30, the product is considered to have failed. This evaluation aim of the simulation case is to assess the reliability level of the product under normal operating conditions (T = 45 °C).

### 4.2. Model Comparison

**Figure 4:**
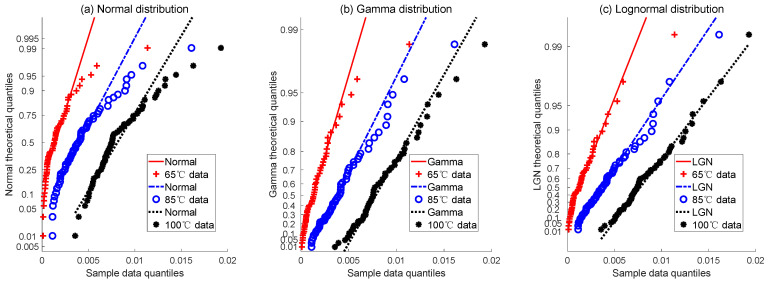
The P-P plots of 3 candidate degradation models for simulation data.

In the modeling of the degradation process for this simulation case, three representative stochastic processes including Wiener, gamma, and lognormal processes are selected for the case analysis of degradation models. In statistics, a P-P plot (probability–probability plot) is a probability graph used to check whether tested data conform to a particular probability distribution. If data conform to the specified distribution, the data will appear to be nearly a straight line [43]. Figure 4 depicts P-P plots for the incremental degradation of the simulated data under normal, gamma, and lognormal distributions. The red solid line, blue dashed line, and black dotted line represent the matching status of the simulated data and distribution at 65 °C, 85 °C, and 100 °C, respectively. From the graph, it can be observed that the P-P plot of the normal distribution deviates slightly from a straight line, while plots for the gamma and lognormal distributions are closer to a straight line. Based on the interpretation of the P-P plot, it suggests that among the three stochastic degradation models, the simulated data may be more in line with the gamma distribution and lognormal distribution. But it is still not possible to directly judge the quality of the models.

**Figure 5:**
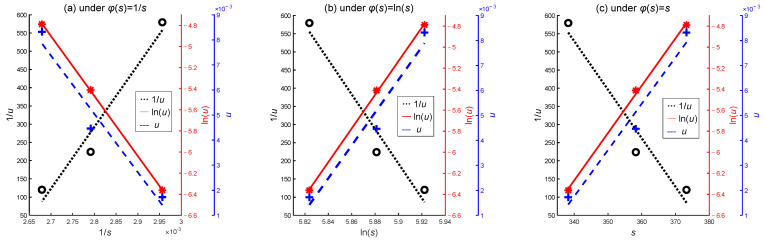
Linear fitting graph of accelerated models for simulation data.

Meanwhile, for the accelerated model of Equation (15), 1/*u*, ln(*u*), and *u* are chosen as the alternative functions of φ(*u*), with 1/*s*, ln(*s*), and *s* as alternative functions of φ(s). This results in nine different combinations of accelerated models. Figure 5 shows a simple linear fitting between different accelerated models of φ(*u*) and φ(s). The value of *u* is calculated using the mean of the degradation rate from the sample data. It is evident that the fitting result of φ(*u*) = ln(*u*) is significantly better than 1/*u* and *u*. There is no apparent difference in the fitting result among 1/*s*, ln(*s*), and *s* for the function φ(*s*). However, due to the limited number of stress levels, it is not possible to make a definitive judgment on the superiority of different stress functions for the accelerated models based on visual observation alone. To simplify the analysis process, avoid an excessive number of combination models, and demonstrate the feasibility of the model averaging evaluation method, three representative accelerated models are selected as candidate accelerated models in this simulation case, representing the Arrhenius, power law, and exponential relations, respectively, shown as

(33) $$\left\{\begin{array}{l} \ln\left(u\right)=a+b/s\;\;\;\;\;\;\mathrm{Arrhenius}\;\mathrm{relation}\\ u=a+b\cdot \ln\left(s\right)\;\;\;\;\;\mathrm{Power}\;\mathrm{law}\;\mathrm{relation}\\ 1/u=a+b\cdot s\;\;\;\;\;\;\;\mathrm{Exponential}\;\mathrm{relation} \end{array}\right..$$

### 4.3. Set of ADT Models

To conduct uncertainty analysis of models for the simulation case, the Wiener process, gamma process, and lognormal process are selected as candidates for the degradation model. Additionally, the Arrhenius relation, power law relation, and exponential relation of Equation (33) are chosen as candidates for accelerated models. The set of ADT models is shown in Table 2, denoted as ***M*** = {*M_v_*}, where *v* = 1,2,…,9. The prior parameters for *M_v_* are ***θ_v_*** = (*a_v_*,*b_v_*,σ*_v_*). The purpose of setting up cross-combinations of models is to examine the influence of different combinations of degradation and accelerated models on the reliability assessment results in the simulation case.

Uniform distributions within a certain interval are set as non-informative prior distributions in this study case, which are shown in Table 3. This helps avoid affecting the calculation result of relative entropy due to significant differences in the choice of prior distributions under different models. In this case, the parameters were iterated 10^5^ times based on sample data, resulting in a set of posterior parameters and degradation data with 10^5^ data points.

### 4.4. Comparison of Prior and Posterior Distribution

For the same sample data, the comparison of prior and posterior distributions under different models can reveal the ability of the ADT Bayesian evaluation method to adjust parameters through different models. In order to analyze the posterior parameters, it is necessary to discard a certain number of samples from the initial iterations of the posterior parameter vector as part of the burn-in phase, and then sample a fixed number of samples from the convergence phase for subsequent calculations. In this case, the last 10,000 samples from the end of the iterations were extracted for analysis as posterior parameter samples for each individual model. 

**Figure 6:**
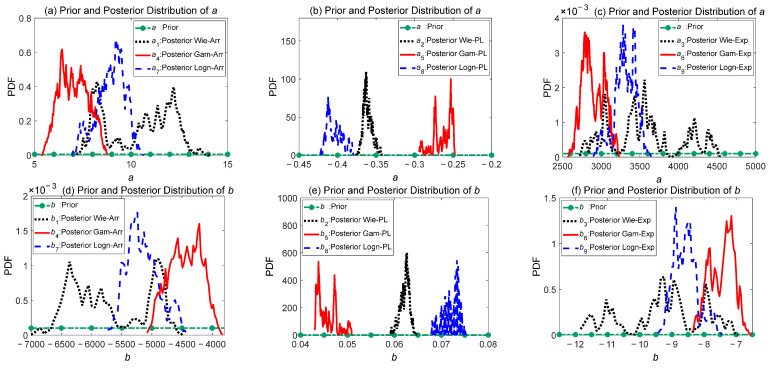

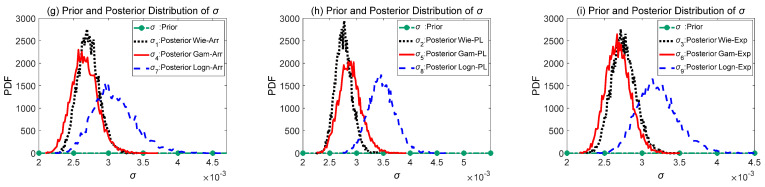
Comparison graph of the prior distributions and posterior distributions for parameters *a*, *b*, and *σ*.

As shown in Figure 6, the parameters coming from the same accelerated model are placed in the same graph, where the green dotted lines represent uniform prior distributions. As the number of iterations increases, it can be seen that the sample data make significant adjustments to the prior distribution, resulting in different posterior distributions of different models that contain model information. In the posterior distributions of the nine different models, the parameter *σ* for all models exhibits a strong normal distribution property. Parameters *a* and *b* do not exhibit distinct distribution characteristics and fluctuate within certain ranges.

In order to compare the posterior parameters with the simulation assumption, the mean, standard deviation, 2.5th percentile, and 97.5th percentile of the posterior distribution for each parameter are provided for analysis. In Table 4, it can be observed that *π*(***θ****|y*) of *M*_1_, *M*_4_, and *M*_7_ are relatively close to the original simulation assumptions. This is because they all utilize the Arrhenius relation as the accelerated model. Although the values of parameters *a* and *b* for *M*_2_, *M*_3_, *M*_5_, *M*_6_, *M*_8_, and *M*_9_ differ significantly from the original assumptions, when plugged into the corresponding accelerated models, the posterior parameters *u* obtained at 65 °C, 85 °C, and 100 °C are relatively similar to the degradation increment values of unit time calculated under the original assumption conditions. Simultaneously, the mean values of the posterior parameter *σ* for all models are consistent with the original assumption conditions. Through the analysis of the posterior distributions, it was found that all three stochastic processes provide reasonably good fits to the sample data. However, there are significant differences in the fits among the accelerated models, which aligns with the conclusions in the model comparison section. 

### 4.5. Calculation of Relative Entropy

**Figure 7:**
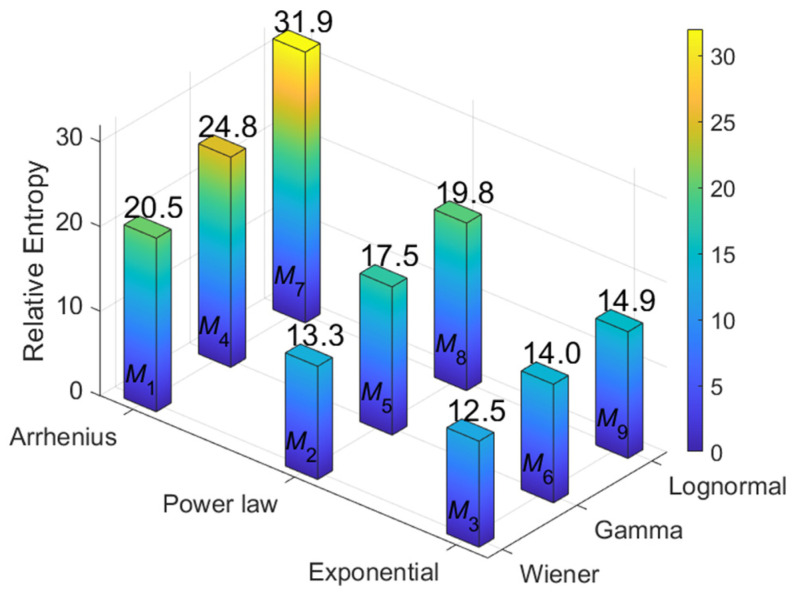
Results of the relative entropy calculation.

Using the same last 10,000 samples from the end of the posterior parameter iterations, the Monte Carlo simulation method and the Laplace–Metropolis algorithm are applied to calculate E***_θ_***[log*f*(*y*|***θ***)] and E***_θ_***[log*m*(*y*)]. The relative entropy results are displayed in Figure 7. The following observations can be obtained:

(1) The relative entropy value of *M*_7_ is the highest of all models, while the degenerate model and accelerated model of *M*_7_ are consistent with the original simulation assumption. From a Shannon information perspective, it represents the maximum IG obtained through *M*_7_ from the sample data.
(2) In this simulation case, the choice of the accelerated model is crucial. Therefore, under the correct Arrhenius relation of the accelerated model, a generally higher value of relative entropy is achieved, while the power law and exponential relation yield relatively lower relative entropy values, which is consistent with the result analysis of Figure 5.
(3) The selection of the degradation model is equally important. The correct lognormal process also provides a higher relative entropy value than other processes for the simulation case, but its advantage is not particularly pronounced. This may be because in situations with limited sample data, the features that align with the lognormal process in the original hypothesis exhibited by simulated data are not very prominent. Therefore, employing other stochastic processes for degradation modeling and analysis can also yield good results.

Based on the relative entropy obtained, weight values of each individual model *M_v_* were calculated through Equation (29), as shown in Table 5. By utilizing these weight values and the MCMC sampling method, the mean $\overset{-}{u}$ of Equation (31) and ${\bar{\sigma }}^{2}$ of Equation (32) after model weight allocation can be obtained. Through $\overset{-}{u}$ and ${\bar{\sigma }}^{2}$, the evaluation results under the model averaging evaluation method can be analyzed.

### 4.6. Result of Model Averaging

**Figure 8:**
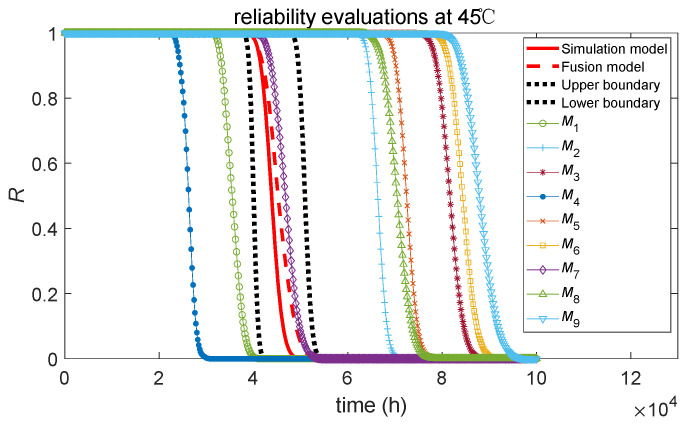
Comparison of reliability evaluation curves.

Figure 8 shows the reliability evaluation curves under normal stress conditions at 45 °C of *M_v_* and the averaging evaluation model. The red solid line represents the reliability evaluation curve of the simulation hypothesis. To some extent, it can represent the actual reliability level of the product to which the simulation data belong. The red dashed line represents the reliability evaluation curve for the Bayesian averaging evaluation method based on relative entropy weights, while the black dotted lines represent the upper and lower boundaries of the 95% confidence interval for reliability under this method. Reliability evaluation curves obtained through individual models of *M*_1_~*M*_9_ are marked curves. The following conclusions can be drawn from the graph:

(1) Among all the reliability evaluation curves for individual evaluation models, the reliability evaluation curve of the *M*_7_ model, which combines the degradation model of the lognormal process with the accelerated model of the Arrhenius relation, closely matches the true reliability assessment curve (red solid line). Therefore, it can be considered that in the case of correct modeling, the Bayesian assessment of ADT through individual models can provide a good estimate of the true product reliability level.
(2) Among the nine individual Bayesian evaluation models, the choice of the accelerated model is crucial. Curves with the same accelerated model exhibit a certain degree of convergence, with the curves for *M*_1_, *M*_4_, and *M*_7_ being similar and *M*_2_, *M*_5_, and *M*_8_ being similar, while *M*_3_, *M*_6_, and *M*_9_ also share similarities.
(3) The reliability evaluation curves based on the accelerated model of the Arrhenius relation for *M*_1_, *M*_4_, and *M*_7_ are relatively close to the true curve, while the reliability evaluation curves based on the accelerated models of the power law relation and exponential relation deviate from the true curve. The analysis reveals that different stochastic processes can effectively describe the simulation data well at a single stress level. However, the limited three stress levels in the simulation case result in significant parameter estimation bias in the accelerated model, leading to substantial deviations in the degradation mean under normal stress conditions at 45 °C.
(4) The reliability evaluation curve obtained by the model averaging method based on relative entropy weights closely matches the true reliability evaluation curve. The upper and lower boundaries of the model averaging evaluation method completely envelop the true reliability evaluation curve, demonstrating the feasibility of the Bayesian averaging evaluation method based on relative entropy.
(5) The reliability evaluation curve of the incorrect models deviates from the true curve; their lesser contribution, due to lower relative entropy weights, minimally affects the evaluation model after averaging. Conversely, the correct models, with higher weights, have a greater influence on the model averaging evaluation method. Through model averaging, the robustness of reliability evaluation is improved. Although both the averaged model and the correct individual model *M*_7_ can effectively obtain the true reliability result in this case, it is very difficult to obtain the true ADT Bayesian model before evaluation in the small-sample evaluation process. Therefore, the Bayesian averaging evaluation method based on relative entropy weights would have a certain advantage.
(6) If the ADT process of a product does not consistently follow a specific evaluation model, then applying the averaging evaluation method proposed in this paper for real-time evaluation of the product’s ADT would be highly meaningful. This approach avoids relying solely on individual models for evaluation. Under the averaging evaluation method considering model uncertainty, as data accumulates, the weight values will change based on the true model properties of the degradation data, thereby continuously approaching the true model and making the evaluation results more reliable.

## 5. Conclusions

Focusing on ADT Bayesian evaluation based on small samples, this article develops a new Bayesian averaging evaluation method for ADT considering model uncertainty based on relative entropy, resulting in some meaningful conclusions.

(1) Drawing from information entropy theory, relative entropy is proposed as a means to evaluate the quality of the ADT Bayesian model. A higher relative entropy value indicates that the model can offer more information gain with the same sample data, suggesting a better fit for the model. Subsequently, a new Bayesian averaging evaluation method for ADT based on relative entropy is developed, demonstrating theoretical feasibility.
(2) Through an illustrative simulation case, a set of simulated data is generated using a lognormal process for the degradation model and an Arrhenius relation for the accelerated model. An uncertainty analysis of models is then conducted, and reliability evaluation curves are obtained under the averaging evaluation method based on relative entropy. The results demonstrate that the proposed method’s evaluation outcomes are consistent with the simulation hypothesis.
(3) Synthesizing the findings from the simulation case study, it is observed that the Bayesian averaging evaluation method based on relative entropy weights can alleviate biases caused by incorrect model selection. It effectively addresses the issue of inaccurate evaluation due to model uncertainty, thus enhancing the robustness of the Bayesian method, which is particularly crucial when dealing with limited sample data.

Furthermore, there are several aspects that warrant further in-depth research and analysis. Firstly, the choice of prior distribution can impact the calculation of relative entropy. In this paper, a simple approach using a uniform distribution as a non-informative prior distribution is employed. However, further in-depth analysis is warranted to examine the influence of different forms of prior distribution on the results of relative entropy. Secondly, the method proposed in this paper is particularly suitable for ADT analysis with limited sample data. From the simulation case study, the differences in relative entropy values among different models are not significant. However, as the degradation data accumulate, the relative entropy weights will gradually favor the true model. How to effectively determine the true model based on relative entropy is also a thought-provoking question. Finally, while this paper employs a simulation case study to validate the feasibility of the proposed method, it is crucial to implement the application of the proposed approach by applying it to more experimental cases in the future.

## Figures and Tables

**Table 1:** Basic information settings of the illustrative simulation case.

Content	Values
Degradation process	Lognormal
Accelerated model	Arrhenius
Simulation parameter ***θ***	*a* = 10, *b* = −5000, *σ* = 0.003
Stress levels (Temperature/°C)	65, 85, 100
Normal stress level (Temperature/°C)	45
Sample size under each stress level	6, 6, 6
Monitor times	10, 10, 10
Failure threshold	30

**Table 2:** Cross-combinations of degradation models and accelerated models.

Model	Arrhenius	Power Law	Exponential
Wiener	*M*_1_	*M*_2_	*M*_3_
Gamma	*M*_4_	*M*_5_	*M*_6_
Lognormal	*M*_7_	*M*_8_	*M*_9_

**Table 3:** Settings of prior distributions.

Prior Distribution	*a*	*b*	*σ*
*π*(***θ***_1_)	unif (−100, 100)	unif (−10,000, 0)	unif (0, 1)
*π*(***θ***_2_)	unif (−100, 100)	unif (−100, 100)	unif (0, 1)
*π*(***θ***_3_)	unif (0, 10, 000)	unif (−100, 100)	unif (0, 1)
*π*(***θ***_4_)	unif (−100, 100)	unif (−10,000, 0)	unif (0, 1)
*π*(***θ***_5_)	unif (−100, 100)	unif (−100, 100)	unif (0, 1)
*π*(***θ***_6_)	unif (0, 10, 000)	unif (−100, 100)	unif (0, 1)
*π*(***θ***_7_)	unif (−100, 100)	unif (−10,000, 0)	unif (0, 1)
*π*(***θ***_8_)	unif (−100, 100)	unif (−100, 100)	unif (0, 1)
*π*(***θ***_9_)	unif (0, 10, 000)	unif (−100, 100)	unif (0, 1)

**Table 4:** Information of posterior distributions.

Posterior Distribution	*a*				*b*				*σ*			
	Mean	Std	2.5%	97.5%	Mean	Std	2.5%	97.5%	Mean	Std	2.5%	97.5%
*π*(***θ***_1_*|y*,*M*_1_)	10.4	1.739	7.77	12.93	−5670	640.6	−6602	−4701	0.0027	0.00016	0.0024	0.003
*π*(***θ***_2_*|y*,*M*_2_)	−0.3609	0.005783	−0.3707	−0.348	0.06264	0.0009843	0.06005	0.06392	0.0028	0.00015	0.0025	0.0031
*π*(***θ***_3_*|y*,*M*_3_)	3573	461.4	2810	4443	−9.253	1.239	−11.59	−7.207	0.0028	0.00016	0.0025	0.0031
*π*(***θ***_4_*|y*,*M*_4_)	7.052	0.7313	5.78	8.414	−4436	267.9	−4934	−3969	0.0027	0.00019	0.0023	0.0031
*π*(***θ***_5_*|y*,*M*_5_)	−0.263	0.01206	−0.2915	−0.2492	0.04567	0.002061	0.04326	0.05049	0.0029	0.00021	0.0025	0.0033
*π*(***θ***_6_*|y*,*M*_6_)	2896	140.1	2666	3172	−7.434	0.3759	−8.174	−6.819	0.0027	0.00017	0.0024	0.003
*π*(***θ***_7_*|y*,*M*_7_)	8.976	0.7286	7.369	10.17	−5139	266.9	−5577	−4553	0.0031	0.0003	0.0026	0.0038
*π*(***θ***_8_*|y*,*M*_8_)	−0.404	0.009927	−0.4178	−0.3821	0.07028	0.001697	0.0683	0.0744	0.0035	0.00025	0.003	0.004
*π*(***θ***_9_*|y*,*M*_9_)	3313	139	2987	3540	−8.563	0.3715	−9.172	−7.696	0.0032	0.00027	0.0027	0.0038

**Table 5:** Weight values of model averaging evaluation method.

{*M_v_*}	*ω_v_*
*M*_1_	0.121
*M*_2_	0.07881
*M*_3_	0.07391
*M*_4_	0.1465
*M*_5_	0.1033
*M*_6_	0.08261
*M*_7_	0.1888
*M*_8_	0.1168
*M*_9_	0.08827

## Data Availability

The data presented in this study are available on request from the corresponding author.
